# Distributions of Cranial Pathologies Provide Evidence for Head-Butting in Dome-Headed Dinosaurs (Pachycephalosauridae)

**DOI:** 10.1371/journal.pone.0068620

**Published:** 2013-07-16

**Authors:** Joseph E. Peterson, Collin Dischler, Nicholas R. Longrich

**Affiliations:** 1 Department of Geology, University of Wisconsin, Oshkosh, Wisconsin, United States of America; 2 Department of Geology and Geophysics, Yale University, New Haven, Connecticut, United States of America; University of Pennsylvania, United States of America

## Abstract

Pachycephalosaurids are small, herbivorous dinosaurs with domed skulls formed by massive thickening of the cranial roof. The function of the dome has been a focus of debate: the dome has variously been interpreted as the product of sexual selection, as an adaptation for species recognition, or as a weapon employed in intraspecific combat, where it was used in butting matches as in extant ungulates. This last hypothesis is supported by the recent identification of cranial pathologies in pachycephalosaurids, which appear to represent infections resulting from trauma. However, the frequency and distribution of pathologies have not been studied in a systematic fashion. Here, we show that pachycephalosaurids are characterized by a remarkably high incidence of cranial injury, where 22% of specimens have lesions on the dome. Frequency of injury shows no significant difference between different genera, but flat-headed morphs (here interpreted as juveniles or females) lack lesions. Mapping of injuries onto a digitial pachycephalosaurid skull shows that although lesions are distributed across the dome, they cluster near the apex, which is consistent with the hypothesis that the dome functioned for intraspecific butting matches.

## Introduction

Pachycephalosauridae is a diverse group of small, herbivorous dinosaurs known from the Late Cretaceous of North America, Asia, and possibly Europe [1], [2]. Their most striking feature is the development of a cranial dome, which is formed by the fusion and thickening of the frontals and parietals, and in some species, peripheral bones of the skull roof. No living animal has a similar morphology, and so the function of this extreme cranial morphology is debated.

There are two primary hypotheses proposed to explain dome function. The first suggests the dome was a display structure [3], and acted either as a sexually selected display or for species recognition [4]. These explanations are problematic because the dome requires a very high investment of material for a display structure, and because the gross similarity in dome shape between different species, as well as extraordinary changes in shape between juveniles and adults [5], [6], would have made the dome relatively ineffective for species recognition [7].

The second hypothesis suggests the dome’s structure served a mechanical function, specifically, that the thickened dome was used in intraspecific agonistic bouts [8]–[11], with pachycephalosaurids butting flanks or heads. The hypothesis that the dome functioned as a weapon is supported by a number of lines of evidence. First, the dome was much better able than structures in any modern head-striker to withstand the high loads that would be imposed by head-butting, and to absorb impact energy that might affect the brain [12], [13]. Second, while no living vertebrate has such an extensive dome, the horns of musk oxen (*Ovibos moschatus*) and Cape Buffalo (*Syncerus caffer*) form a domed structure that is used for head-to-head ramming [14]. The analogy in structure implies analogy in function, and structure-behavior correlations place pachycephalosaurids among the best extant head-stikers [13]. However, trace evidence of behavior in the form of pathology would provide further support for structure-behavior correlations.

More recently, pathologies have been identified in pachycephalosaurid domes [11], [15]. Pathologies are of interest because injuries tend to occur in parts of the anatomy that are used frequently and/or subject to unusually high strains; insofar as pathologies reflect the wear-and-tear experienced by an animal, they can provide a record of the behavior of the living organism [15]–[18]. Peterson and Vittore [15] suggested that the lesions on the dorsal surface of a dome of *Pachycephalosaurus wyomingensis* resulted from infection following an injury. This diagnosis was based on the presence of irregular lesion floors, smooth margins, and internal rarefaction zones consistent with osteomyelitis–infection of bone and marrow–following trauma to the external covering to the dome [15]. Although the composition of overlying soft tissue on pachycephalosaurid domes is not known, it has been hypothesized to have been minimal [12], and would lead to a higher likelihood of infection. Characteristics of chronic osteomyelitis include thick, sclerotic, irregular bone, elevated periosteum and chronic draining sinus tracts [19]. Chronic osteomyelitis can develop following trauma to the bone itself or spread from an adjacent soft-tissue infection, resulting in deep-penetrating lesions [15], [19].

The presence of pathologies in pachycephalosaurid domes would support the hypothesis that domes were used as weapons. Although scores of pachycephalosaurid domes are known, few lesions have been reported. In this context, the question arises whether the few pathologies known from pachycephalosaurid skulls are simply the result of chance, or whether injuries to the dome are more common than would be expected, suggesting a that the injuries result from the behavior of the animals. Furthermore, different combat styles enable different predictions about the distribution of injuries. Flank-butting would be expected to produce relatively few injuries because the dome would primarily impact the muscular sides of an opponent; injuries would also tend to occur on the side of the dome if the head was swung laterally. Head-on ramming would produce a high frequency of injuries that would be concentrated at the apex of the dome.

To test the hypothesis that frontoparietal domes were used for combat, we examined the distribution and frequency of pathologies in skulls and skull domes of Pachycephalosauridae; a high frequency of pathologies would indicate frequent damage to the dome. Based on the frequency of injuries in extant head-striking vertebrates, this correlation would support a combat function for domes. Rather than focusing only on domes with injuries, we attempted to examine all available domes, injured and uninjured, to analyze the frequency and spatial distributions of the lesions.

## Materials and Methods

**Figure 1:**
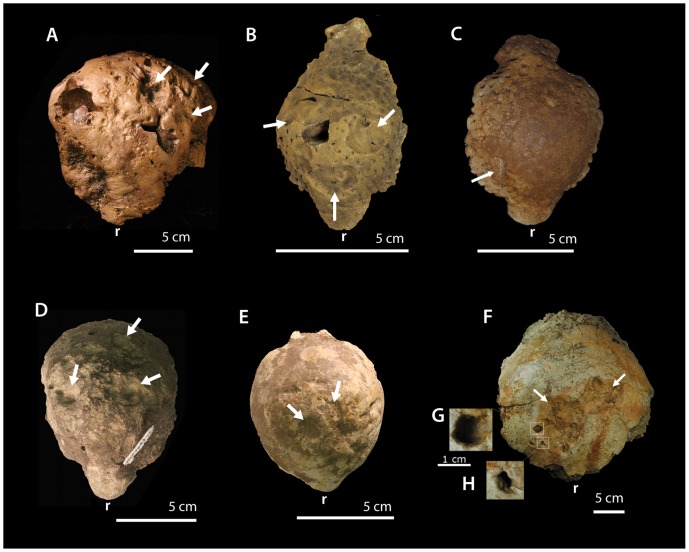
Selected pathological pachycephalosaurid specimens. (A) TMP 72.27.01, *Gravitholus albertus* in dorsal view of erosive lesions; (B) TMP 1992.2.3, *Stegoceras validum* in dorsal view with arrows denoting dorsal lesions; (C) TMP 2011.012.0009, *Stegoceras validum* in dorsal view with arrow denoting dorsal lesion; (D) TMP 1997.99.2, an unidentified pachycephalosaurid in dorsal view with arrows denoting lesions; (E) AMNH 0044, *Sphaerotholus buchholtzae*, in dorsal view with arrows denoting dorsal lesions; (F) BMRP 2001.4.1, *Pachycephalosaurus wyomingensis* in dorsal view with arrows denoting large depression features and high magnification of deep erosive lesions (G, H). Rostral portion of the frontal denoting “r”.

To investigate the frequency and spatial distribution of lesions in frontoparietal domes, 109 domes from over 14 species were examined for pathologies and surface irregularities (Table 1; Table S1). Specimens were studied from the holdings of 22 vertebrate paleontology collections (Text S1) by study of the original specimens, casts, and high-resolution photographs from the literature when necessary (Figure 1). Access to examined specimens and casts was permitted by housing institutions by either on-site examination or specimen loans.

**Table 1:** Analyzed pachycephalosaur genera and frequency of pathologic frontoparietals.

Genus	Injured Domes	Examined Domes
*Amtocephale*	1	1
*Colepiocephale*	2	6
*Dracorex*	0	1
*Goyocephale*	0	1
*Gravitholus*	1	1
*Hansseusia*	1	7
*Homalocephale*	0	1
*Pachycephalosaurus*	2	9
*Prenocephale*	0	1
*Sphaerotholus*	5	22
*Stegoceras*	8	41
*Stygimoloch*	0	5
*Texacephale*	1	2
*Tylocephale*	0	1
*Pachycephalosauridae indet.*	3	10
	24	109

Institutional restrictions prevented coring or thin-sectioning for histological analyses; however computed tomography (CT) scans were conducted on available domes that possessed depressions. The scans were performed with Aquilion Toshiba 64-slice CT scanners at Rockford Memorial Hospital in Rockford, IL and Aurora Healthcare Systems in Oshkosh, WI. Scans were conducted at settings for medical diagnoses of bone pathology (135 kV, 300 mA, 0.5 mm pixel resolution, and 0.5 mm thickness). The relatively high density and thickness of some specimens resulted in poor resolution of CT images. The raw CT data are archived at the Burpee Museum of Natural History and the Royal Tyrrell Museum of Palaeontology. A subset of the domes examined for this study was also digitized into 3D models using a NextEngine Desktop 3D Scanner and processed with ScanStudio HD Pro (NextEngine) (Figures S1–S5).

Lesions were differentially diagnosed based on CT data and the presence of gross pathological characteristics consistent with osteological damage, such as irregular-shaped lesion surfaces, remodeling, and rounded margins of lesion [15], [19]. In order to differentiate lesions from taphonomic artifacts, comparisons were conducted with bovid skulls possessing lesions resulting from trauma, bone resorption, and taphonomic alteration (i.e. insect modification and bone weathering).

### Lesion Distribution

**Figure 2:**
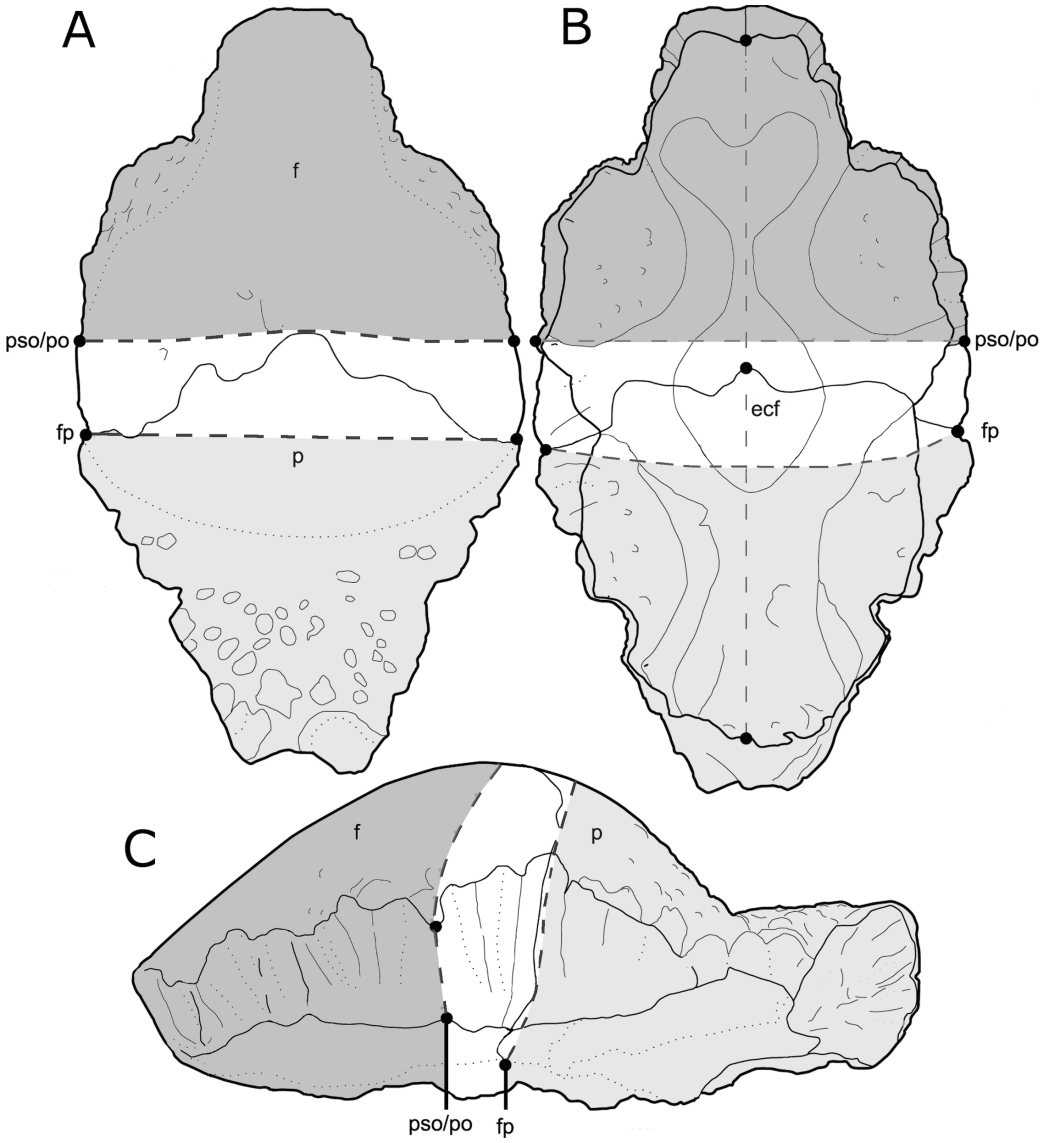
Schematic diagram illustrating the morphometric landmark-defined regions of lesion distribution in dorsal (A), ventral (B), and left lateral (C) views. Abbreviations: f, frontal; p, parietal; fp, frontoparietal suture; pso, posterior supraorbital; po, postorbital; ecf Modified from Schott et al., 2011 with permission.

To analyze the distribution of cranial abnormalities in pachycephalosaurid crania, lesions were characterized and tallied for their presence within one of three “zones” characterized by homologous morphometric landmarks on frontoparietal domes [6]. These three zones include:

1. Frontal Zone- defined by the prefrontal-frontal suture to the rostral-most point of the frontoparietal suture;
2. Sutural Zone- defined by the area enclosed by the posterior supraorbital and postorbital sutural surfaces and the rostral-most point of the frontoparietal suture;
3. Parietal Zone-defined by the caudal-most point of the frontoparietal suture to the parietal-squamosal suture (Figure 2).

Concave lesions were plotted in relationship to morphometric landmarks onto a 3D model of *Stegoceras validum* (UALVP2) using the morphometric software Landmark (IDAV) and categorized based on their location.

In order to investigate variation in lesion distributions with respect to dome shape, the specimens were grouped as partially- or fully-domed [20], [21]. Taxa that possessed a frontoparietal dome with a fully developed parietal were considered fully domed (e.g. *Sphaerotholus*, *Pachycephalosaurus*, and other members of Pachycephalosaurinae). Similarly, taxa with a partially developed and incorporated parietal were considered partially-domed (e.g. *Stegoceras*, *Colepiocephale*, and *Gravitholus*). A chi-square test was used to determine whether a statistically significant relationship existed between dome shape and distribution of pathologies.

### Survey of Injuries in Extant Bovids

In order to explore the distribution of healed fractures and lesions on known “head-butting” animals, a survey was conducted of 30 skeletons of extant bovids housed in the University of Wisconsin Zoology Museum, Madison, WI and the Field Museum of Natural History, Chicago, IL. The genera were chosen on their agonistic behaviors as a hypothetical behavioral model for pachycephalosaurids. These included flank-butting domestic goats (*Capra*), clashing or “head-butting” Bighorn and Dall sheep (*Ovis*) and “head-shoving” American bison (*Bison*) [14]. Many of the bovid specimens were former zoo and farm specimens. Female specimens were included in this study; while males are more likely to engage in intraspecific combat, female bovids also engage in intrasexual competition [22].

Skeletons were analyzed for injuries in four regions: 1. cranial skeleton (crania and mandibles), 2. cervical skeleton (atlas, axis, and cervical vertebrae), 3. thoracic skeleton (pectoral girdle, thoracic ribs, and thoracic vertebrae), and 4. pelvic skeleton (pelvic girdle and sacral vertebrae). Different combat styles were predicted to produce different frequencies of injury on different regions. With their broadside and “flank-butting” behaviors [14], [23], *Capra* skeletons were predicted to exhibit a high frequency of injury in their thoracic and lumbar skeletons. Broad-horned ruminants, such as domestic bulls (*Bos*) and American bison (*Bison*) frequently engage in low-impact “head-shoving” and “horn-wrestling”, where injuries were expected on the thoracic and lumbar skeletons, due to falling and rolling during shoving bouts [23]. The characteristic clashing or “head-butting” behavior of extant Bighorn and Dall sheep (*Ovis*), similar to the behaviors hypothesized for pachycephalosaurids, were predicted to produce injuries on the crania, at the point of impacts [14], [23], [24]. Percent abundance was calculated for the presence of injuries in each region for the three genera. Due to relatively small samples of available pathological specimens, further statistical analysis was unable to be determined.

## Results

### Lesion Characteristics and Computed Tomography

Lesions were identified and diagnostically differentiated based on the criteria previously outlined for lesion identification in pachycephalosaurids [15] (Table 2). Pit-like depressions can result from a variety of taphonomic processes, such as bites, insect borings, and fluvial erosion. However, evidence of healing shows lesions were endured before death.

**Table 2:** Hypothesized mechanisms of dome modification, and predictions made for each hypothesis.

Hypothesis	Pit-like depressions	Presence in thick bones	Healing	Specific to Pachycephalosaurs	Specific to frontoparietals	Specific to adults
Insect borings	X	–	–	–	–	–
Theropod/Crocodylia feeding traces	X	X	–	–	–	–
Fluvial erosion/abrasion	X	X	–	–	–	–
Non-traumatic bone resorption	X	–	X	–	–	–
Infectious disease	X	X	X	X	–	–
Trauma-induced osteomyelitis	X	X	X	X	X	X

Although a number of different hypotheses could potentially account for damage to the frontoparietal domes, infection resulting from trauma is the only one that would account not only for the damage seen, but also the existence of healing and the restriction of infection to the dorsal part of the dome in mature pachycephalosaurids.

Cranial lesions have been described in a variety of dinosaurs. Lesions appearing in the squamosal fenestrae of numerous specimens of chasmosaurine ceratopsids have been attributed to non-traumatic bone resorption [25]. However, bone remodeling or “punched out lesions” (POLs) described in ceratopsians usually occur in thin regions of the squamosal, on both internal and external bone surfaces, and the lesions exhibit smooth surfaces [25]. The relatively massive construction of frontoparietal domes is inconsistent with the POLs seen in thin ceratopsian squamosals and parietals. This suggests that non-traumatic bone resorption is not a likely source of the depressions.

Lesions resulting from agonistic behavior in pachycephalosaurids were expected to occur in relatively high frequency on the dorsal surface of the frontoparietal dome. Furthermore, injuries would be expected in specimens representing later ontogenetic stages rather than low-domed or flat-headed juveniles. The lesions on pachycephalosaurid frontoparietals occur on the dorsal surface of the dome on specimens of later ontogenetic stages; lesions are not present on low-domed or flat-headed juveniles. As such, the combat hypothesis and its predictions best fit the observations and is the most likely etiology (Table 2). Furthermore, lesions were characterized by the presence of thick sclerotic, irregular bone surfaces, which are commonly associated with chronic osteomyelitis [19].

**Figure 3:**
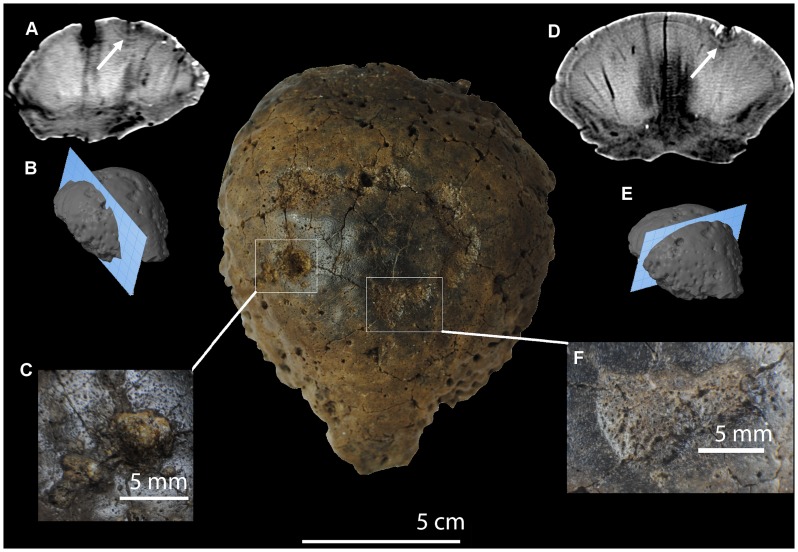
Computed tomography images from TMP 79.14.853, *Hansseusia sternbergi*. Scale bar equals 5 cm. Sagittal section (A, B) of frontoparietal with arrow annotating low-density region immediately ventral to dorsal lesion (C); Coronal section (D, E) of frontoparietal dome illustrating low-density region immediately ventral to dorsal lesion (F).

Computed tomography of four domes (BMR P.2001.4.5, TMP 79.14.853, TMP 2010.005.0008, and TMP 92.2.3) showed regions of chronic changes to the surface of the concave lesions that have smooth and rounded margins, suggestive of healing (Figures 3C, F). These lesions vary in size, ranging from over four cm to less than five mm in width. Furthermore, the expected irregularity of the floor of the lesions is well demonstrated in pathologic specimens (Figures 3C, F). These irregular floors show a varying thickness of higher-density bone over a rarefied zone, which does not exist at the same depth elsewhere in the specimens, consistent with woven osseous remodeling and overlying new bone. A few small rounded concavities penetrate the bone surface at the periphery of the larger defects which is consistent with chronic osteomyelitis (Figures 3A–B, D–E). These characteristics suggest the lesions are of traumatic etiology with superimposed, ongoing infection.

### Frequency and Distribution of Lesions among Fully- and Partially-domed Taxa

**Figure 4:**
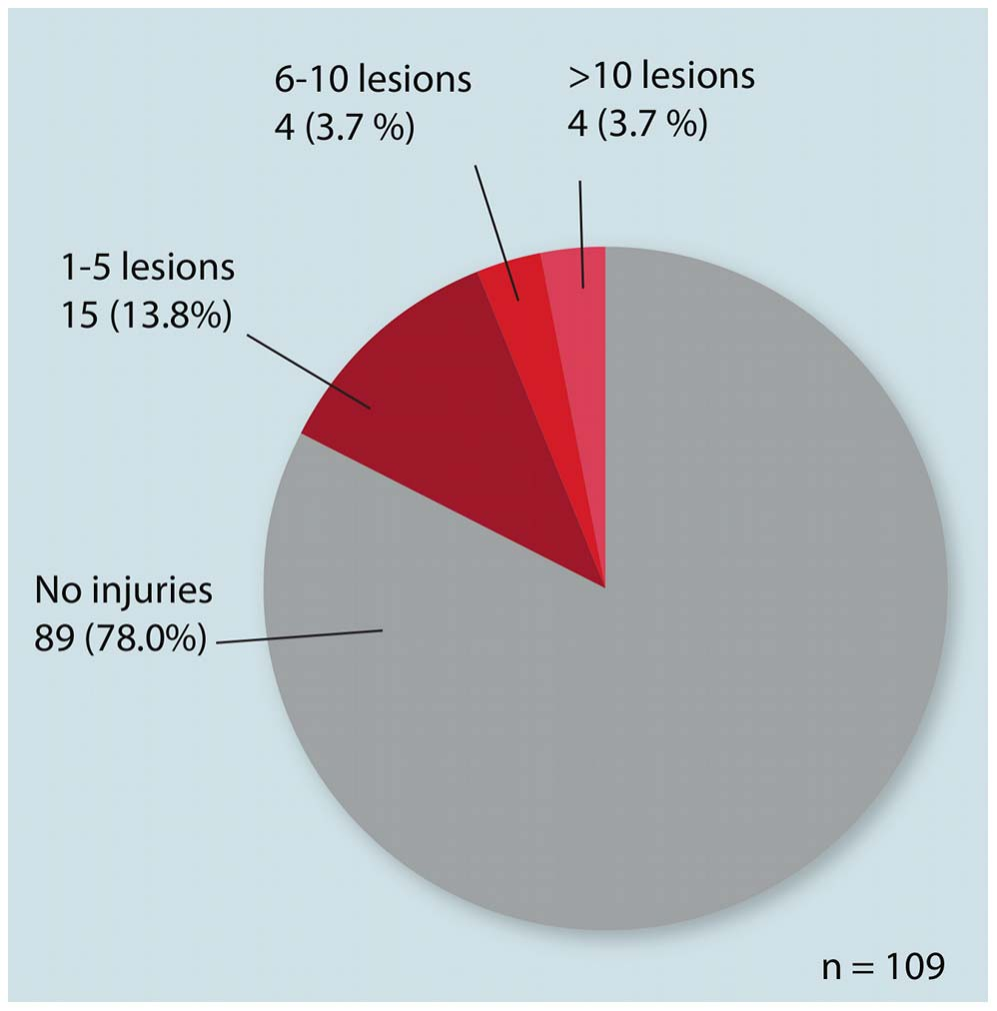
Frequency of pathologic frontoparietal domes of the total sample size (n = 109).

**Figure 5:**
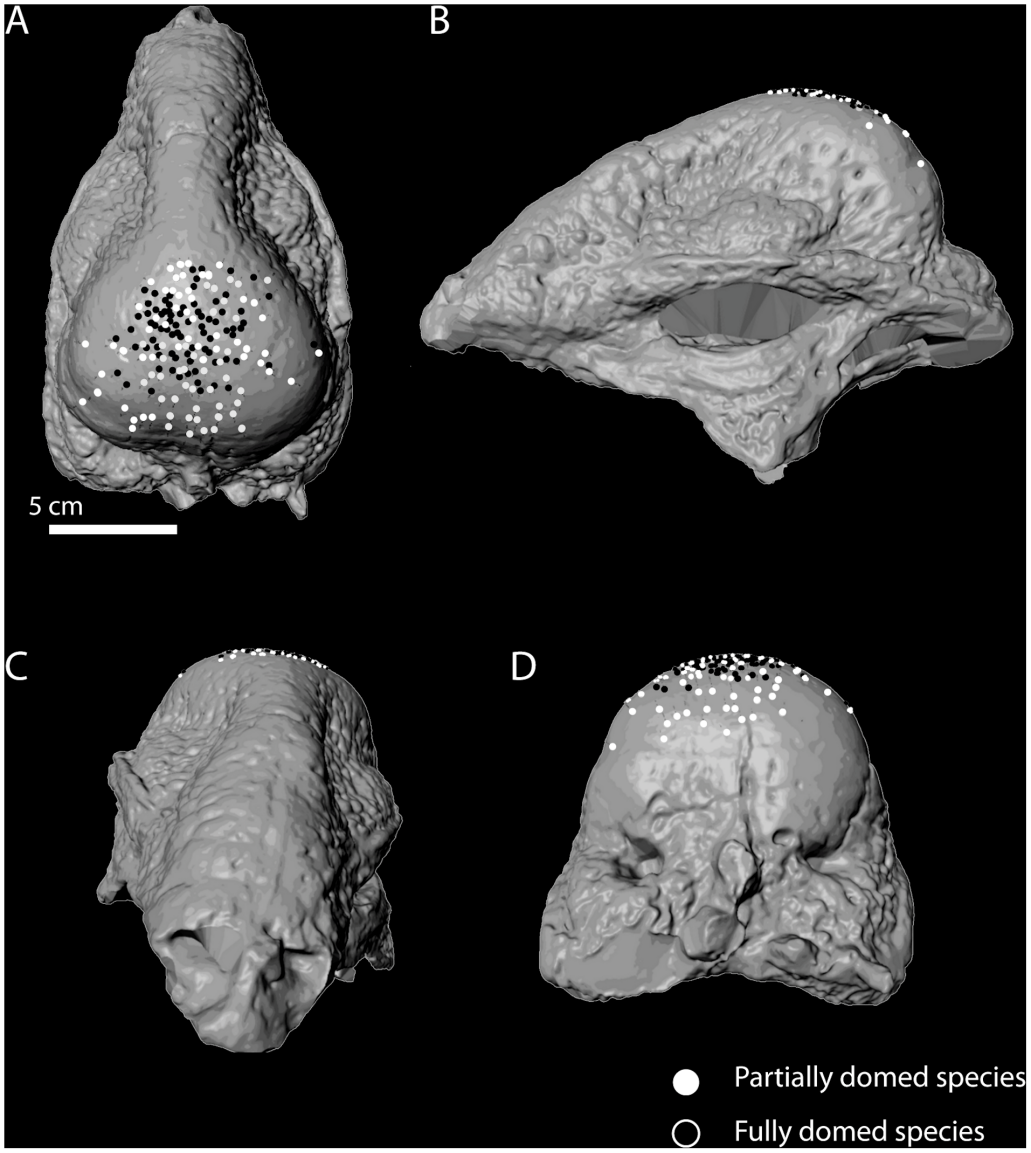
Distribution of total observed lesions on pachycephalosaurid cranial model (UALVP 2, *Stegoceras validum*). Skull in dorsal (A), left lateral (B), rostral (C), and caudal views (D).

**Figure 6:**
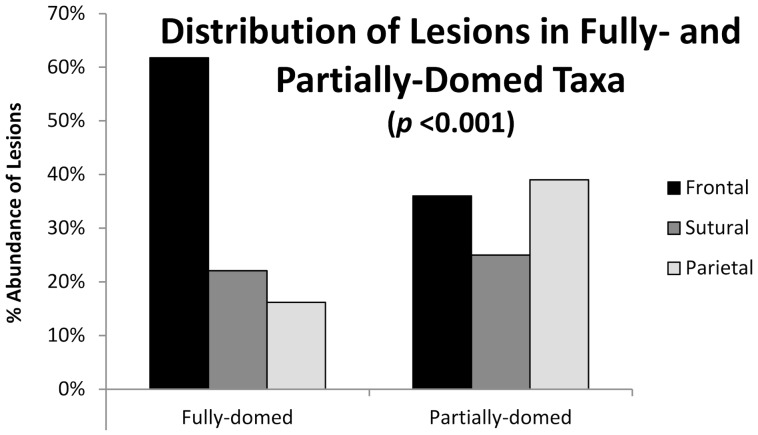
Distribution of lesions among frontal, sutural, and parietal regions in high- and low-domed specimens.

Of the 109 analyzed specimens, 24 specimens (22%) from at least 9 species possess depressions on the dorsal surface that show features consistent with pathologies (Figure 4, Table 3). Percent abundance was calculated for the distribution of lesions within the frontal, sutural, and parietal zones of domes (Figures 5, 6). Fully-domed specimens possessed a higher frequency of lesions on the frontal zone (over 63%) compared to partially-domed specimens, which possess a lower frequency of frontal lesions (35% frontal).

**Table 3:** Pathologic frontoparietal dome specimens and distribution of lesions.

			Lesion Distribution			
Taxon	Specimen	Frontal	Sutural	Parietal	H:L Ratio	Doming Group
*Amtocephale gobiensis*	MPC-D 100/1203	7	5	3	0.21	NA
*Colepiocephale lambei*	TMP 2000.57.01	5	2	0	0.17	Partial
*Colepiocephale lambei*	TMP 1992.88.01	3	1	1	0.36	Partial
*Gravitholus albertus*	TMP 72.27.01	4	15	20	0.36	Partial
*Hanssuesia sternbergi*	TMP 1979.14.853	6	4	0	0.29	Full
Pachycephalosauridae indet.	TMP 1997.99.2	5	2	5	0.3	Partial
Pachycephalosauridae indet.	TMP 87.36.364	4	0	0	0.49	Partial
Pachycephalosauridae indet.	TMM 42532-3	2	1	2	0.53	Partial
*Pachycephalosaurus wyomingensis*	BMR P2001.4.5	23	7	8	0.26	Full
*Pachycephalosaurus wyomingensis*	DMNS 469	5	0	0	0.31	Full
*Sphaerotholus brevis*	AMNH 1697	1	0	0	0.14	Full
*Sphaerotholus brevis*	CMN 121	1	0	0	0.27	Full
*Sphaerotholus brevis*	CMN 8819	0	1	1	0.28	Full
*Sphaerotholus buchholtzae*	TMP 1987.113.3	1	0	0	0.41	Full
*Sphaerotholus sp.*	AMNH 0044	5	3	2	0.29	Full
*Stegoceras sp.*	UALVP 8502	0	0	2	0.21	Partial
*Stegoceras sp.*	TMP 1992.2.3	6	0	0	0.33	Partial
*Stegoceras validum*	AMNH 1697	1	0	0	0.24	Partial
*Stegoceras validum*	AMNH 1699	1	0	0	0.24	Partial
*Stegoceras validum*	TMP 2011.012.0009	1	0	0	0.29	Partial
*Stegoceras validum*	TMP 1998.93.125	1	1	1	0.3	Partial
*Stegoceras validum*	TMP 2001.602.0015	0	0	4	0.3	Partial
*Stegoceras validum*	UALVP 5	3	2	4	0.54	Partial
*Texacephale langstoni*	LSUMNS 20010	0	1	0	0.38	Partial

Results of a Chi-square Test of Independence (0.05 significance level) for the distribution of lesions among fully- and partially-domed taxa show a significant difference (<0.005). Thus, we can reject the null hypothesis that lesions are distributed randomly with respect to dome shape (Table S2). The total frequency of injuries differs between partially-domed species (25%) and fully-domed species (18%) but the difference in frequency is not significant using a Chi-Square test.

### Comparisons with Extant Bovids

**Figure 7:**
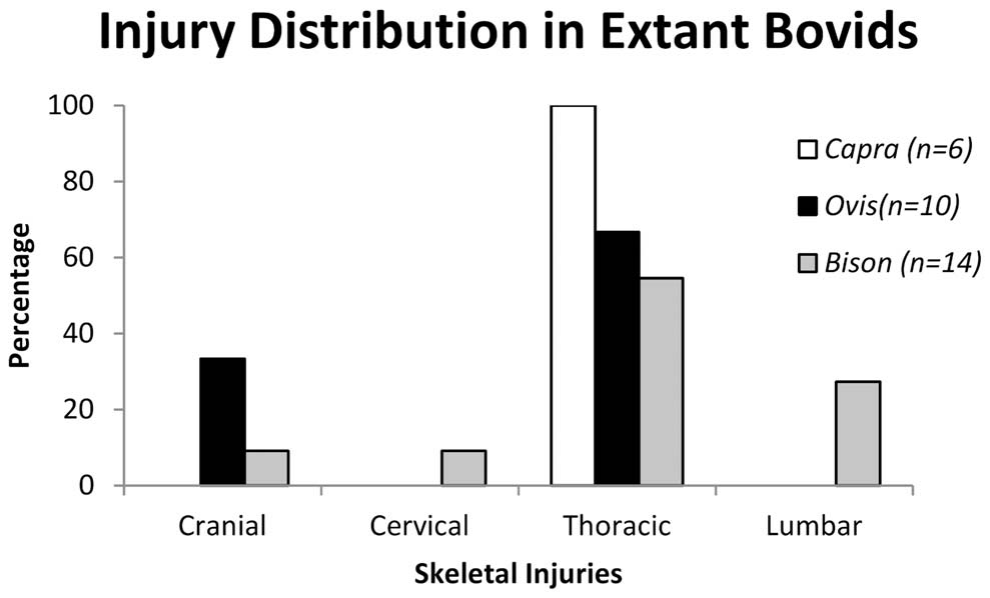
Distribution and frequency of injuries on the postcranial skeleton of extant bovids; *Capra*, *Ovis*, and *Bison*.

An examination of injuries in of extant bovids that are known for intraspecific aggression yielded different distributions of injuries among the three genera (Figure 7, Table S3). While the total number of injuries was low, their distributions correspond with the specific agonistic combat style observed in each genus [8], [23].

**Figure 8:**
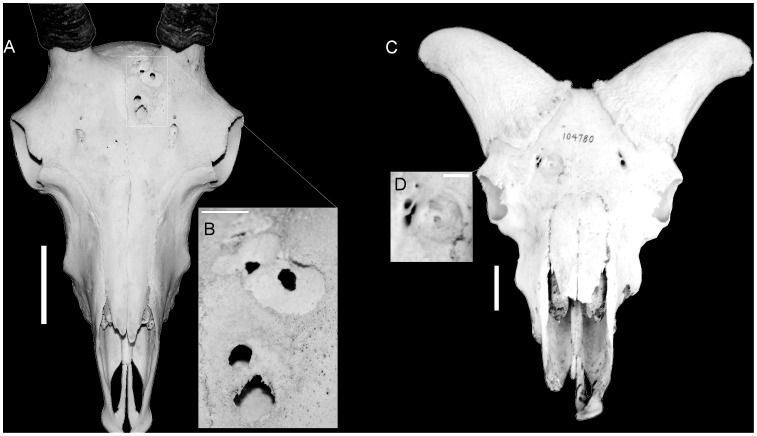
Frontal lesions identified on skulls of *Ovis*. *Ovis canadensis* (A) illustrating smaller lesions clustering around main depression (B), and *Ovis dalli* (C) illustrating irregular lesion floors (D). Scale bars for A and C equal 5 cm. Scale bars for B and D equal 10 mm.

**Figure 9:**
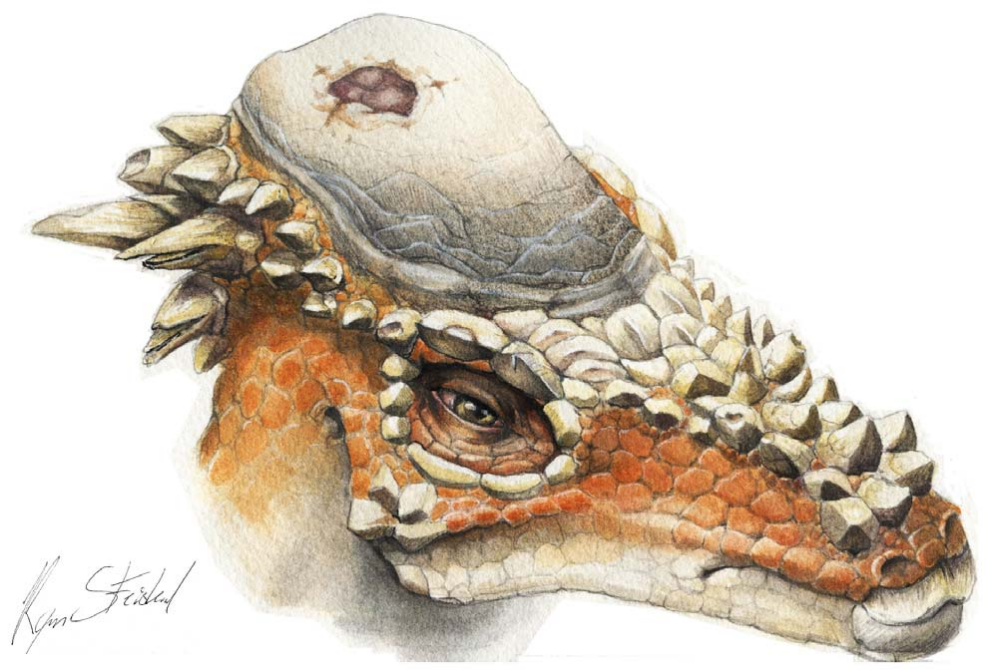
Reconstruction of *Pachycephalosaurus wyomingensis* with cranial lesion.

As expected, injuries present in goats (*Capra*) were to found occur exclusively on the thoracic skeleton (ribs and thoracic vertebrae); corresponding with the broadside and lateral “flank-butting” agonistic behaviors of domestic goats [14], [26]. Meanwhile, the injuries present on *Bison* included the expected thoracic and lumbar injuries. However, injuries were also identified on the cranial skeleton of *Bison*, likely resulting from an individual becoming injured while breaking away from a bout, as previously documented in field observations [27]. The injuries identified in skeletons of *Ovis* included the predicted cranial injuries, but also include injuries to the thoracic skeleton. These injuries were commonly fractured and healed ribs that may have resulted from occasional broadside impacts or falling as a result of clashing, as documented in field observations [27]. Lesions present on the crania in *Ovis* resulting from intraspecific combat (Figures 8A, B) show characteristics consistent with cortical damage, such as irregular-shaped lesion surfaces, remodeling, and smooth, rounded margins of lesions [19]. The lesions present in the crania of *Ovis* and other head-butting bovids are similar to the injuries observed in pachycephalosaurids, and suggest a similar behavioral origin (Figure 9).

## Discussion

Pachycephalosaurid domes exhibit a remarkably high incidence of pathology, where approximately one-fifth of all domes have lesions that are consistent with osteomyelitis. Osteomyelitis can result from a number of different processes, but the most likely one in this context is trauma to the skull, with damage to the tissue overlying the skull leading to an infection of the bone tissue. The high frequency of pathology seen in pachycephalosaurids is, therefore, consistent with the hypothesis that the dome was employed in intraspecific combat. It is also difficult to explain in any other context. Meanwhile, the absence of pathology in flat-headed pachycephalosaurids- which presumably represent juveniles or females [11] is consistent with the hypothesis that these injuries are the result of intraspecific combat; agonistic bouts among extant bovids occur relatively more frequently among mature males [14]. Interestingly, the frequency of injury appears to be comparable in different species, despite the fact that they vary in dome architecture and size, and existed at different times. As such, this may suggest a phylogenetically constrained pattern.

A high incidence of pathology is also seen in other dinosaurs thought to engage in combat. For instance, injuries occur in the tail spines of the armored dinosaur *Stegosaurus* [17] and in the frill of *Triceratops* [18], A high frequency of injury is also seen among birds that engage in intraspecific combat, including steamer ducks (*Tachyeres* spp.) [28], the Rodriguez Island Solitaire (*Pezophaps solitarius*) [29], and the extinct Jamaican club-winged ibis (*Xenicibis*) [30]. However the frequency of injury is less in other dinosaurs (9.8% in *Stegosaurus*, and 14% in *Triceratops*) than in pachycephalosaurs. This implies that the frequency and/or intensity of combat in pachycephalosaurids was markedly higher.

The high frequency of pathology is remarkable when one considers the high fitness cost that must have been imposed. A number of specimens studied here, for example *Gravitholus* (TMP 72.27.01), exhibit extensive osteomyletis with limited healing, and thus may have died from their injuries. Even if these infections were not fatal, battling an infection of the bone would have presumably made it more difficult to forage, evade predators, and compete for mates, therefore exacting a high toll on fitness. This cost would be imposed on top of the already considerable cost of growing, maintaining, and carrying a massive bony structure atop the skull. For natural selection to favor the growth of such a dome and combat leading to these injuries, the dome must have contributed a substantial benefit to fitness, one that greatly outweighed its costs.

In this context, the most likely explanation for the dome is that it is the product of sexual selection–selection not for the survival of the individual, but for the ability to acquire a mate and to reproduce (e.g. [31], [32]). Pachycephalosaurids presumably used the dome to fight other members of the same species, either to secure access to mates, to secure territories, or both. In extant mammals, such as Bighorn sheep, males typically fight other males for access to females [11]. However, this is not the only possibility. For instance, among birds, the Northern Jacana (*Jacana spinosa*) has an unusual breeding system in which males provide all parental care, and females are polyandrous [33]. Females, armed with spike-like wing spurs, fight aggressively against each other to establish territories containing multiple males. Intraspecific combat also occurs in monogamous birds. In the extinct solitaire *Pezophaps*, both males and females were reported to cooperate to defend territories against other members of the species [29]. Thus, the evolution of a cranial dome and intraspecific combat does not necessarily imply a polygamous mating system. By analogy with living birds and mammals, pachycephalosaurids could conceivably have been polygamous, monogamous, or polyandrous.

The frequency of frontoparietal injuries in domed and flat-headed morphs may also suggest variation in frontoparietal function over the course of ontogeny. No flat-headed pachycephalosaurids were found to exhibit pathologies; injuries are limited to individuals with domed skulls. This implies that the development of the dome was associated with the individual engaging in intraspecific combat.

The proposed function of the dome also has implications for understanding its histology. Frontoparietal domes are composed of a unique form of fibrolamellar bone [34]. Histologic examination of frontoparietal domes reveals this particular form of fibrolamellar bone closely resembles periosteal bone, but lacks canaliculi [35]. Fibrolamellar bone is also present in the parietosquamosal shields of ceratopsians; in both occurrences fibroblasts rapidly deposit bone during remodeling [35], [36]. Fibroblasts are also important during bone healing processes [37], and exhibit predictable osteological responses to trauma and infection. Lesions from trauma on the jugals and parietosquamosal shields of ceratopsians have also been identified and extensively described [18], [25]. Given the rapidity of fibrolamellar remodeling, such tissues may have been selected in structures in need of frequent remodeling and healing.

**Figure 10:**
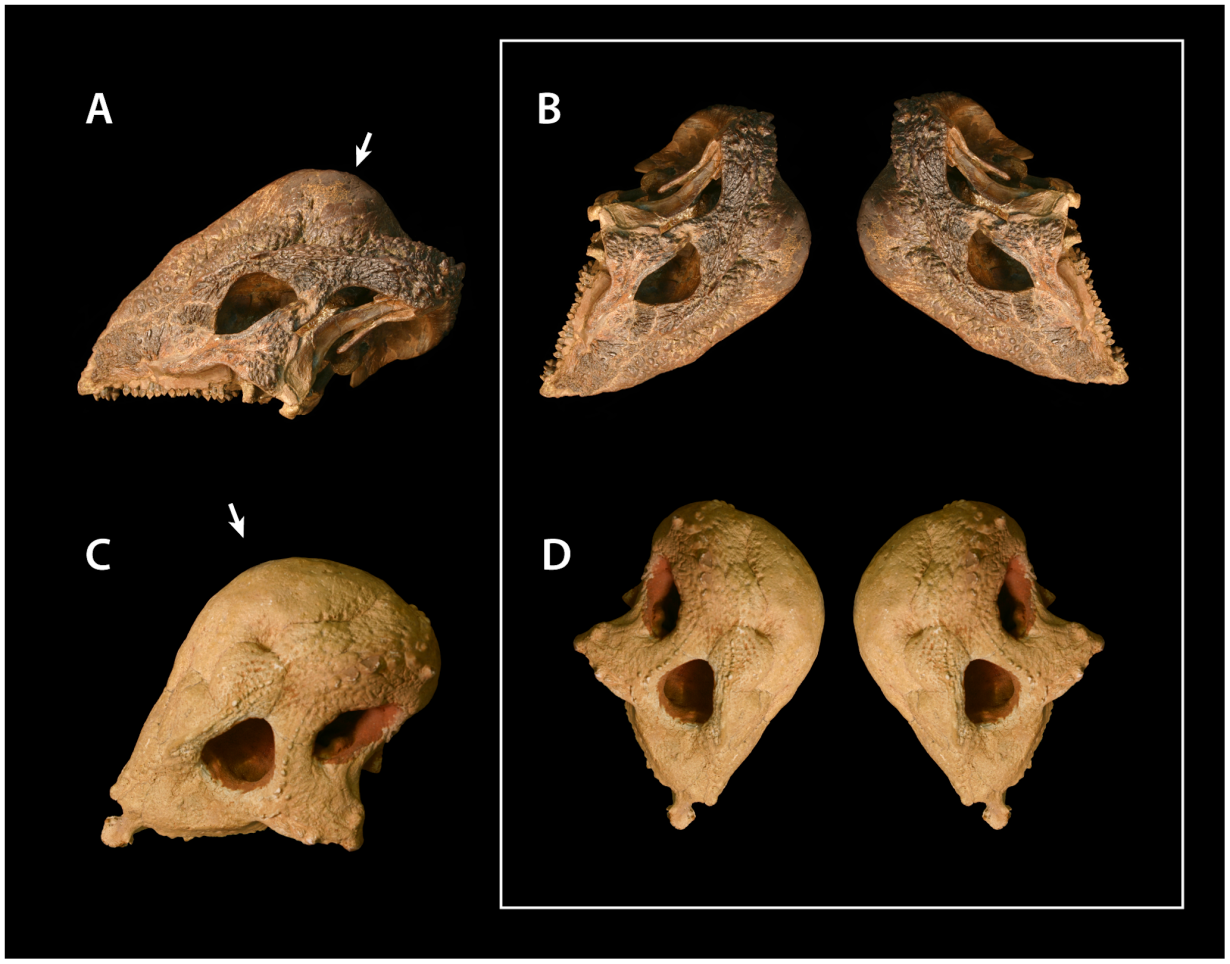
Apex of frontoparietal dome of UA2 (*Stegoceras validum*) (A), and cranial orientation during combat (B); Apex of frontoparietal dome of ZPAL MgD-I/104 (*Prenocephale prenes*) (C), and cranial orientation during combat (D).

**Figure 11:**
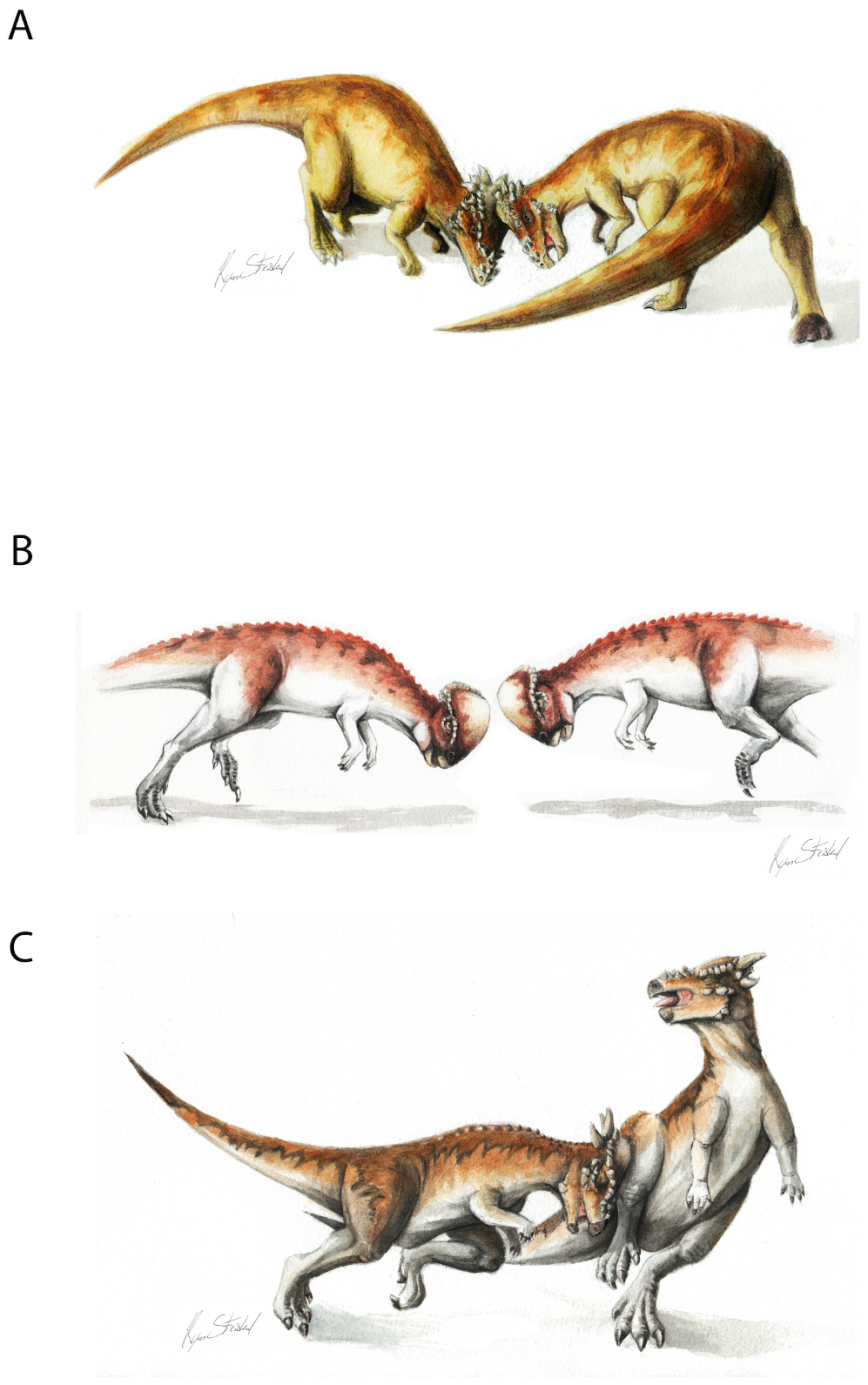
Hypothetical head-to-head interactions among pachycephalosaurids. (A) *Bison*-like head-shoving in large, broad-domed specimens such as *Pachycephalosaurus wyomingensis;* (B) *Ovis*-like clashing in *Prenocephale prenes*; (C) *Capra*-style broadside butting in high-domed and large-horned specimens such as subadult *Pachycephalosaurus* (“*Stygimoloch*” and “*Dracorex*”).

**Figure 12:**
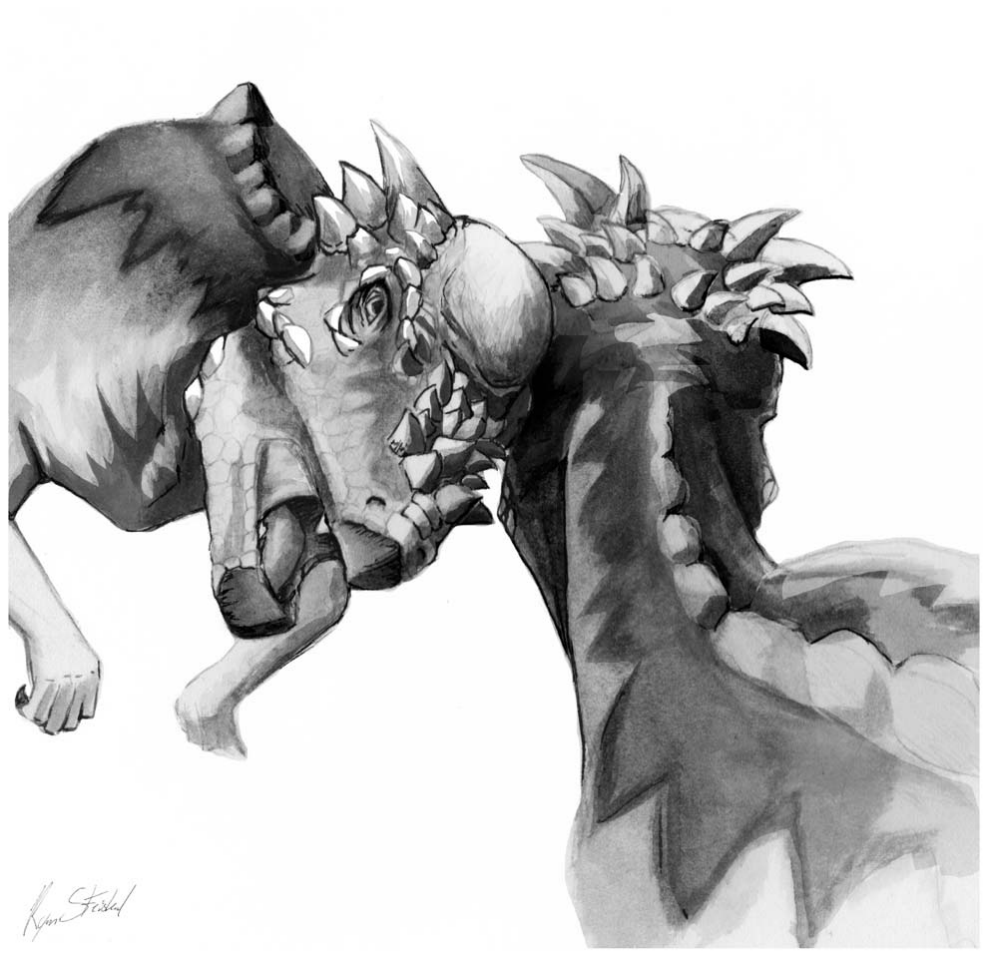
Hypothetical head-to-body interactions among pachycephalosaurids. *Giraffa*-style necking and parietal clashing in high-domed and large-horned specimens such as subadult *Pachycephalosaurus* (“*Stygimoloch*” and “*Dracorex*”).

Comparisons with injuries in extant bovids illustrate the variation in injury and lesion distribution related to behavior (Figures 7, 8A, B) and suggest that the distribution of injuries in extinct animals can therefore be similarly used to infer behavior in extinct taxa. Frontoparietal domes have long been hypothesized to have functioned as battering rams for head-to-head collisions or flank-butting during agonistic bouts similar to that observed in extant artiodactyls [8], [38]. The functional capability of such behavior has been supported by finite element modeling of the structural capabilities of frontoparietal domes [12], [13]. However, agonistic behaviors in extant artiodactyls vary considerably with horn and cranial morphology [14], [23], and the distribution of injuries in extant bovids illustrates this variation. Similarly, the variation in frontoparietal dome shape (whether ontogenetic or phylogenetic) suggests a variety of functions for frontoparietal domes, including a variety of agonistic functions (Figure 10). The distribution of injuries on frontoparietal domes supports this hypothesis; the high frequency of lesions on the frontal regions of fully-domed taxa may suggest head-shoving or head-butting behaviors similar to those observed in extant *Bison*, *Ovis*, *Ovibos*, or *Syncerus* (Figures 11A, B). Alternatively, the relatively equal distribution of injuries on partially-domed specimens may suggest more complex agonistic interactions including “dome/horn-wrestling” similar to the behaviors observed in *Capra*, *Oreamnos*, or *Aepyceros* (Figures 11C, 12). Post-cranial injuries on pachycephalosaurids are unknown, due to a lack of available pachycephalosaurid post-cranial skeletons. However, based on these results, analogous post-cranial injuries to ribs and pelvic girdles are predicted and may offer further insight into specific behaviors.

The variation in doming among pachycephalosaurids, as well as the occurrence of elaborate horn morphologies in some taxa, may suggest a broad ontogenetic and phylogenetic variation in dome function. The relationship between lesion morphology and the highly clustered presence of lesions on the frontal and parietal regions of domes suggests that each region was subject to considerable osteological necrosis due to trauma and possibly secondary infection following agonistic interactions. These results do not provide unequivocal evidence for any specific behavior and frontoparietal dome function in pachycephalosaurids; based on comparisons with other vertebrates that possess extreme cranial structures, domes likely served multiple functions [39]. However, the strong relationship between lesion distribution and the structure of frontoparietal domes [12], [13] supports the hypothesis that pachycephalosaurids used their unique cranial structures for agonistic functions.

Although the evidence suggests that the dome functioned as a weapon, other possibilities cannot be ruled out. Goodwin and Horner [4] proposed that frontoparietal domes and accompanying ornamentations created a brightly-colored visual display, much like the striking displays in cassowaries and toucans, and were not utilized as a weapon in agonistic bouts. However, most cranial structures in extant vertebrates are used for multiple functions [39], [40]. Given this, evidence for combat does not rule out a display function. Indeed, conspicuous weapons are inherently effective as displays. Consider that sexual display features are selected for because they are honest signals of fitness; a peacock’s elaborate tail is a signal that its bearer is able to secure the resources to build the tail while fighting off diseases and parasites and evading predators. A conspicuous weapon is an honest signal as well: it communicates its owner’s ability to fight for mates or territory. Bighorn sheep, for example, exhibit a threat behavior in which they lower the horns into position to butt an opponent [41]. Displaying a weapon and the willingness to use it may often be enough to settle a dispute, without resorting to actual combat. It follows that while not all display structures are weapons (the peacock’s tail, for instance), weapons can be display structures.

In conclusion, an examination of over 100 pachycephalosaurid domes reveals an extraordinary high frequency of pathology. The structure of these pathologies is consistent with osteopathic myelitis, infection of the bone. Given the distribution of the lesions–they are frequent in pachycephalosaurids, they are confined to adult pachycephalosaurids, and they are concentrated on the apex of the dome, near its thickest point–these lesions are best explained as the result of trauma incurred by intraspecific head-butting matches, leading to abrasion and then to infection. Along with the massive construction of the dome, this suggests that the dome of pachycephalosaurids was primarily used to fight for mates, territory, or both. The pachycephalosaurian dome, therefore, and its remarkable history of injuries, hints at a rich, if conflict-filled, social life for these animals of which we still know little.

## Supporting Information

Figure S1 **3D PDF model of TMP72.27.01.**
(PDF)

Figure S2 **3D PDF model of BMRP2001.4.5.**
(PDF)

Figure S3 **3D PDF model of TMP1992.2.3.**
(PDF)

Figure S4 **3D PDF model of TMP2011.012.0009.**
(PDF)

Figure S5 **3D PDF model of TMP79.14.853.**
(PDF)

Table S1 **Frontoparietal specimens used in study.**
(DOCX)

Table S2 **Chi-square results of comparisons between doming and lesion distributions.**
(DOCX)

Table S3 **Injuries in extant bovids skeletons.**
(DOCX)

Text S1 **Institutional abbreviations.**
(DOCX)
