# Supplementary material for: Distributions of Cranial Pathologies Provide Evidence for Head-Butting in Dome-Headed Dinosaurs (Pachycephalosauridae)
Source: PLoS One. 2013 Jul 16;8(7):e68620. doi: 10.1371/journal.pone.0068620 (PMC3712952; doi:10.1371/journal.pone.0068620)
Supplement: Figure S3 — 3D PDF model of TMP1992.2.3. (PDF) [file pone.0068620.s003.pdf]

TMP1992.2.3

Stegoceras sp.

25th January 2012

temp.u3d
