# Supplementary material for: Distributions of Cranial Pathologies Provide Evidence for Head-Butting in Dome-Headed Dinosaurs (Pachycephalosauridae)
Source: PLoS One. 2013 Jul 16;8(7):e68620. doi: 10.1371/journal.pone.0068620 (PMC3712952; doi:10.1371/journal.pone.0068620)
Supplement: Table S1 — Frontoparietal specimens used in study. (DOCX) [file pone.0068620.s006.docx]

Supporting Table S1: Frontoparietal specimens used in study.

| **Taxon** | **Specimen** | **Pathological** | **Doming** | **Reference** |
| --- | --- | --- | --- | --- |
| *Amtocephale gobiensis* | MPC-D 100/1203 | 1 | NA | Mahito Watabe and Sullivan, 2011 |
| *Colepiocephale lambei* | CMN 8818 | 0 | Partial | NRL pers. obs. |
| *Colepiocephale lambei* | ROM 3632 | 0 | Partial | NRL pers. obs. |
| *Colepiocephale lambei* | TMP 1970.02.01 | 0 | Partial | NRL pers. obs. |
| *Colepiocephale lambei* | TMP 1992.88.01 | 1 | Partial | NRL pers. obs. |
| *Colepiocephale lambei* | TMP 2000.57.01 | 1 | Partial | NRL pers. obs. |
| *Colepiocephale lambei* | UALVP 31471 | 0 | Partial | NRL pers. obs. |
| *Dracorex wogwartsia* | TCMI 2004.17.1 | 0 | NA | Bakker et al., 2006 |
| *Goyocephale lattimorei* | GI SPS 100/1501 | 0 | NA | Perle et al., 1982 |
| *Gravitholus albertus* | TMP 72.27.01 | 1 | Partial | JEP pers. obs. |
| *Hanssuesia sternbergi* | CMN 192 | 0 | Full | Sullivan, 2003 |
| *Hanssuesia sternbergi* | CMN 38079 | 0 | Full | Sullivan, 2003 |
| *Hanssuesia sternbergi* | CMN 8817 | 0 | Full | Sullivan, 2003 |
| *Hanssuesia sternbergi* | CMN 9952 | 0 | Full | JEP pers. obs. |
| *Hanssuesia sternbergi* | CMN 9148 | 0 | Full | Sullivan, 2003 |
| *Hanssuesia sternbergi* | TMP 1979.14.853 | 1 | Full | JEP pers. obs. |
| *Hanssuesia sternbergi* | TMP 1987.36.363 | 0 | Full | JEP pers. obs. |
| *Homalocephale calathorcercos* | GI SPS 100/1201 | 0 | NA | Perle et al., 1982 |
| *Pachycephalosaurus wyomingensis* | AMNH 1687 | 0 | Full | JEP pers. obs. |
| *Pachycephalosaurus wyomingensis* | AMNH 1771 | 0 | Full | JEP pers. obs. |
| *Pachycephalosaurus wyomingensis* | BMR P2001.4.5 | 1 | Full | JEP pers. obs. |
| *Pachycephalosaurus wyomingensis* | DMNH 15416 | 0 | Full | JEP pers. obs. |
| *Pachycephalosaurus wyomingensis* | DMNS 469 | 1 | Full | JEP pers. obs. |
| *Pachycephalosaurus wyomingensis* | NS.1559.02 | 0 | Full | JEP pers. obs. |
| *Pachycephalosaurus wyomingensis* | UWGM NS.1559.02 | 0 | Full | JEP pers. obs. |
| *Pachycephalosaurus wyomingensis* | USNM 358114 | 0 | Full | JEP pers. obs. |
| *Pachycephalosaurus wyomingensis* | VRD 13 | 0 | Full | Horner and Goodwin, 2009 |
| *Prenocephale prenes* | ZPAL MgD-I/104 | 0 | Full | JEP pers. obs. |
| *Sphaerotholus brevis* | CMN 121 | 1 | Full | NRL pers. obs. |
| *Sphaerotholus brevis* | AMNH 1697 | 1 | Full | JEP pers. obs. |
| *Sphaerotholus brevis* | CMN 12351 | 0 | Full | NRL pers. obs. |
| *Sphaerotholus brevis* | CMN 1423 | 0 | Full | NRL pers. obs. |
| *Sphaerotholus brevis* | CMN 8819 | 1 | Full | NRL pers. obs. |
| *Sphaerotholus brevis* | CMN 8830 | 0 | Full | NRL pers. obs. |
| *Sphaerotholus brevis* | TMP 85. 36.292 | 0 | Full | Schott 2011 |
| *Sphaerotholus brevis* | TMP 85.43.68 | 0 | Full | Schott 2011 |
| *Sphaerotholus brevis* | TMP 87.50.29 | 0 | Full | Sullivan, 2003 |
| *Sphaerotholus breivs* | TMP 91.36.265 | 0 | Full | Schott 2011 |
| *Sphaerotholus breivs* | TMP 99.55.121 | 0 | Full | Schott 2011 |
| *Sphaerotholus breivs* | UALVP 47278 | 0 | Full | NRL pers. obs. |
| *Sphaerotholus breivs* | USNM 537766 | 0 | Full | NRL pers. obs. |
| *Sphaerotholus breivs* | UALVP 8508 | 0 | Full | NRL pers. obs. |
| *Sphaerotholus buchholtzae* | LACM 15342 | 0 | Full | NRL pers. obs. |
| *Sphaerotholus buchholtzae* | LACM 64000 | 0 | Full | NRL pers. obs. |
| *Sphaerotholus buchholtzae* | TMP 1987.113.3 | 1 | Full | NRL pers. obs. |
| *Sphaerotholus edmontonense* | CMN 8831 | 0 | Full | NRL pers. obs. |
| *Sphaerotholus edmontonense* | CMN 8832 | 0 | Full | NRL pers. obs. |
| *Sphaerotholus goodwini* | NMMNH P-27403 | 0 | Full | Williamson and Carr, 2002 |
| *Sphaerotholus sp.* | AMNH 0044 | 1 | Full | JEP pers. obs. |
| *Sphaerotholus sp.* | TMP 2010.005.0008 | 0 | Full | JEP pers. obs. |
| *Stegoceras sp.* | CMN 8818 | 0 | Partial | Williamson and Carr, 2002 |
| *Stegoceras sp.* | TMP 70.02.01 | 0 | Partial | JEP pers. obs. |
| *Stegoceras sp.* | TMP 1980.16.752 | 0 | Partial | JEP pers. obs. |
| *Stegoceras sp.* | TMP 1982.36.1 | 0 | Partial | JEP pers. obs. |
| *Stegoceras sp.* | TMP 1983.209.3 | 0 | Partial | JEP pers. obs. |
| *Stegoceras sp.* | TMP 1992.2.3 | 1 | Partial | JEP pers. obs. |
| *Stegoceras sp.* | TMP 2007.020.0003 | 0 | Partial | JEP pers. obs. |
| *Stegoceras sp.* | UALVP 8502 | 1 | Partial | JEP pers. obs. |
| *Stegoceras sp.* | UALVP 8503 | 0 | Partial | JEP pers. obs. |
| *Stegoceras sp.* | UALVP 8505 | 0 | Partial | JEP pers. obs. |
| *Stegoceras sp.* | UCMP 130051 | 0 | Partial | Goodwin, 1990 |
| *Stegoceras validum* | AMNH 1697 | 1 | Partial | This study |
| *Stegoceras validum* | AMNH 1699 | 1 | Partial | This study |
| *Stegoceras validum* | AMNH 5450 | 0 | Partial | Schott et al., 2011 |
| *Stegoceras validum* | CMN 1108 | 0 | Partial | This study |
| *Stegoceras validum* | CMN 138 | 0 | Partial | Schott et al., 2011 |
| *Stegoceras validum* | CMN 2369 | 0 | Partial | Sullivan, 2003 |
| *Stegoceras validum* | CMN 515 | 0 | Partial | Sullivan, 2003 |
| *Stegoceras validum* | CMN 8816 | 0 | Partial | Sullivan, 2003 |
| *Stegoceras validum* | NMMNH P-33983 | 0 | Partial | Sullivan and Lucas, 2006 |
| *Stegoceras validum* | MOR 1179 | 0 | Partial | This study |
| *Stegoceras validum* | ROM 53555 | 0 | Partial | Schott et al., 2011 |
| *Stegoceras validum* | CMN 38428 | 0 | Partial | JEP pers. obs. |
| *Stegoceras validum* | ROM 803 | 0 | Partial | This study |
| *Stegoceras validum* | TMP 1967.010.0003 | 0 | Partial | This study |
| *Stegoceras validum* | TMP 1975.011.005 | 0 | Partial | This study |
| *Stegoceras validum* | TMP 1981.16.145 | 0 | Partial | This study |
| *Stegoceras validum* | TMP 1983.67.1 | 0 | Partial | This study |
| *Stegoceras validum* | TMP 84.5.1 | 0 | Partial | This study |
| *Stegoceras validum* | TMP 1986.146.2 | 0 | Partial | This study |
| *Stegoceras validum* | TMP 1992.36.286 | 0 | Partial | This study |
| *Stegoceras validum* | TMP 1998.93.125 | 1 | Partial | This study |
| *Stegoceras validum* | TMP 1998.93.79 | 0 | Partial | This study |
| *Stegoceras validum* | TMP 99.62.1 | 0 | Partial | Sullivan, 2003 |
| *Stegoceras validum* | TMP 2000.26.01 | 0 | Partial | Sullivan, 2003 |
| *Stegoceras validum* | TMP 2001.602.0015 | 1 | Partial | This study |
| *Stegoceras validum* | TMP 2003.12.252 | 0 | Partial | This study |
| *Stegoceras validum* | TMP 2011.012.0009 | 1 | Partial | This study |
| *Stegoceras validum* | UALVP 2 | 0 | Partial | JEP pers. obs. |
| *Stegoceras validum* | UALVP 5 | 1 | Partial | JEP pers. obs. |
| *Stegoceras validum* | UCMZ(VP)2008.001 | 0 | Partial | Schott et al., 2011 |
| *Stygimoloch spinifer* | MPM 7111 | 0 | Full | Griffen et al., 1988 |
| *Stygimoloch spinifer* | MPM 8111 | 0 | Full | Horner and Goodwin, 2009 |
| *Stygimoloch spinifer* | NS.1559.01 | 0 | Full | JEP pers. obs. |
| *Stygimoloch spinifer* | UCMP 119433 | 0 | Full | Horner and Goodwin, 2009 |
| *Stygimoloch spinifer* | UCMP 147063 | 0 | Full | Goodwin et al., 1998 |
| *Texacephale langstoni* | LSUMNS 20010 | 1 | Partial | Longrich et al., 2010 |
| *Texacephale langstoni* | LSUMNS 20012 | 0 | Partial | Longrich et al., 2011 |
| *Tylocephale gilmorei* | ZPAL MgD-I/105 | 0 | NA | Maryanska & Osmolska, 1974 |
| Pachycephalosauridae indet. | CMM V87-1 | 0 | Partial | JEP pers. obs. |
| Pachycephalosauridae indet. | NMMNH P-30067 | 0 | Partial | Williamson and Carr, 2002 |
| Pachycephalosauridae indet. | ROM 2962 | 0 | Partial | Sullivan, 2003 |
| Pachycephalosauridae indet. | TMM 42532-1 | 0 | Partial | Lehman, 2010. |
| Pachycephalosauridae indet. | TMM 42532-2 | 0 | Partial | Lehman, 2010. |
| Pachycephalosauridae indet. | TMM 42532-3 | 1 | Partial | Lehman, 2010. |
| Pachycephalosauridae indet. | TMP 1981.19.232 | 0 | Partial | JEP pers. obs. |
| Pachycephalosauridae indet. | TMP 1997.99.2 | 1 | Partial | JEP pers. obs. |
| Pachycephalosauridae indet. | TMP 84.121.21 | 0 | Partial | JEP pers. obs. |
| Pachycephalosauridae indet. | TMP 87.36.364 | 1 | Partial | JEP pers. obs. |
