# Supplementary material for: Distributions of Cranial Pathologies Provide Evidence for Head-Butting in Dome-Headed Dinosaurs (Pachycephalosauridae)
Source: PLoS One. 2013 Jul 16;8(7):e68620. doi: 10.1371/journal.pone.0068620 (PMC3712952; doi:10.1371/journal.pone.0068620)
Supplement: Table S2 — Chi-square results of comparisons between doming and lesion distributions. (DOCX) [file pone.0068620.s007.docx]

Supporting Table S2: Chi-square results of comparisons between doming and lesion distributions.

|  | **Fully-Domed** | **Partially-Domed** | **Total** |
| --- | --- | --- | --- |
| **Frontal Zone** | 42 | 36 | 79 |
| **Sutural Zone** | 15 | 25 | 40 |
| **Parietal Zone** | 11 | 39 | 50 |
| **Total** | 68 | 100 | 168 |
|  |  |  |  |
|  | **Fully-Domed** | **Partially-Domed** |  |
| **Frontal Zone** | 31.57143 | 46.42857 |  |
| **Sutural Zone** | 16.19048 | 23.80952 |  |
| **Parietal Zone** | 20.2381 | 29.7619 |  |
|  |  |  |  |
| **Chi Sq**. | 3.444732 | 2.342418 |  |
|  | 0.087535 | 0.059524 |  |
|  | 4.216919 | 2.867505 |  |
|  |  |  |  |
| **Chi Sq Sum** | 13.01863 |  |  |
| **Degrees of Freedom** | 2 |  |  |
| **Probability** | 0.00148922 |  |  |
|  |  |  |  |
