# Supplementary material for: Distributions of Cranial Pathologies Provide Evidence for Head-Butting in Dome-Headed Dinosaurs (Pachycephalosauridae)
Source: PLoS One. 2013 Jul 16;8(7):e68620. doi: 10.1371/journal.pone.0068620 (PMC3712952; doi:10.1371/journal.pone.0068620)
Supplement: Table S3 — Injuries in extant bovids skeletons. (DOCX) [file pone.0068620.s008.docx]

Supporting Table S3: Injuries in extant bovids skeletons.

| COUNTS | Cranial | Cervical | Thoracic | Lumbar | TOTAL |
| --- | --- | --- | --- | --- | --- |
| *Capra (n=6)* | 0 | 0 | 5 | 0 | 5 |
| *Ovis(n=10)* | 2 | 0 | 4 | 0 | 6 |
| *Bison (n=14)* | 1 | 1 | 6 | 3 | 11 |
|  |  |  |  |  |  |
|  |  |  |  |  |  |
| *PERCENT* | Cranial | Cervical | Thoracic | Lumbar |  |
| *Capra (n=6)* | 0 | 0 | 100 | 0 |  |
| *Ovis(n=10)* | 33.33333 | 0 | 66.66667 | 0 |  |
| *Bison (n=14)* | 9.090909 | 9.090909 | 54.54545 | 27.27273 |  |
