# Supplementary material for: Distributions of Cranial Pathologies Provide Evidence for Head-Butting in Dome-Headed Dinosaurs (Pachycephalosauridae)
Source: PLoS One. 2013 Jul 16;8(7):e68620. doi: 10.1371/journal.pone.0068620 (PMC3712952; doi:10.1371/journal.pone.0068620)
Supplement: Text S1 — Institutional abbreviations. (DOCX) [file pone.0068620.s009.docx]

Supporting Text S1 - Institutional abbreviations.

American Museum of Natural History (AMNH) New York City, NY

Burpee Museum of Natural History (BMR) Rockford, IL

Carter County Museum (CCM) Ekalaka, MT

Canada Museum of Nature (CMN) Ottawa, ON, Canada

Denver Museum of Nature and Science (DMNS) Denver, CO

Paleontological Center, Mongolian Academy of Sciences (formerly Section of Paleontology and Stratigraphy of the Geological Institute, Mongolian Academy of Sciences) (GI SPS) Ulaanbaatar, Mongolia

Los Angeles County Museum (LACM) Los Angeles, CA

Louisiana State University Museum of Natural Sciences (LSMNS) Baton Rouge, LA

Mongolian Paleontological Center (MPC) Ulaanbataar, Mongolia

Milwaukee Public Museum (MPM) Milwaukee, WI

New Mexico Musuem of Natural History (NMMNH) Albuquerque, NM

Royal Ontario Museum (ROM) Toronto, ON, Canada

The Children’s Museum of Indianapolis (TCMI) Indianapolis, Indiana

Texas Memorial Museum (TMM) Austin, TX

Royal Tyrrell Museum of Paleontology (TMP) Drumheller, Alberta, Canada

Vertebrate Paleontology Collection University of Alberta (UALVP) Edmonton, Alberta, Canada

University of California Museum of Paleontology (UCMP) Berkeley, CA

University of Calgary, Museum of Zoology (UCMZ) Calgary, AB

University of Wisconsin Madison Geological Museum (UWGM) Madison, WI

Sierra College (VRD) Rocklin, CA

Palaeozoolocial Institute of the Polish Academy of Science (ZPAL) Warsaw, Poland
